# The role of photorespiration in plant immunity

**DOI:** 10.3389/fpls.2023.1125945

**Published:** 2023-02-01

**Authors:** Xiaotong Jiang, Berkley J. Walker, Sheng Yang He, Jianping Hu

**Affiliations:** ^1^ Michigan State University-Department of Energy Plant Research Laboratory and Department of Plant Biology, Michigan State University, East Lansing, MI, United States; ^2^ Howard Hughes Medical Institute and Department of Biology, Duke University, Durham, NC, United States

**Keywords:** photorespiration, immunity, reactive oxygen species, photorespiratory metabolites, defense hormones

## Abstract

To defend themselves in the face of biotic stresses, plants employ a sophisticated immune system that requires the coordination of other biological and metabolic pathways. Photorespiration, a byproduct pathway of oxygenic photosynthesis that spans multiple cellular compartments and links primary metabolisms, plays important roles in defense responses. Hydrogen peroxide, whose homeostasis is strongly impacted by photorespiration, is a crucial signaling molecule in plant immunity. Photorespiratory metabolites, interaction between photorespiration and defense hormone biosynthesis, and other mechanisms, are also implicated. An improved understanding of the relationship between plant immunity and photorespiration may provide a much-needed knowledge basis for crop engineering to maximize photosynthesis without negative tradeoffs in plant immunity, especially because the photorespiratory pathway has become a major target for genetic engineering with the goal to increase photosynthetic efficiency.

## Introduction

**Figure 1:**
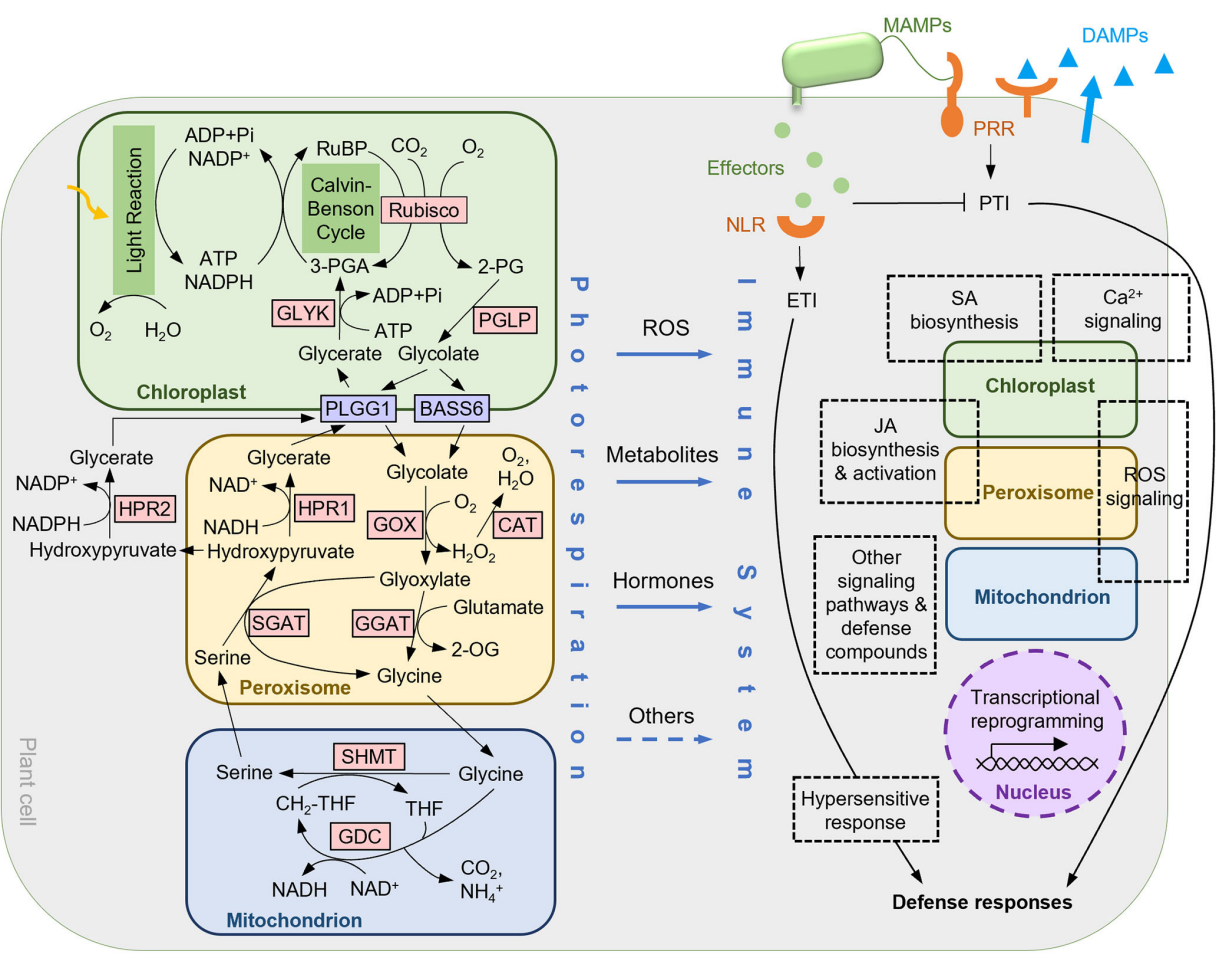
A working model for the connections between photorespiration and plant immunity. ROS, photorespiratory metabolites, defense hormones, and possibly other mechanisms connect the photorespiratory pathway to key components of the immune network. See main text for detailed information of the photorespiratory pathway and plant immunity, as well as mechanisms/potential mechanisms for their connections. Overlaps between some subcomponents of the immune response network and the photorespiratory organelles indicate the involvement of the particular organelles. 2-OG, 2-oxoglutarate; 2-PG, 2-phosphoglycolate; 3-PGA, 3-phosphoglycerate; BASS6, bile acid sodium symporter 6; CAT, catalase; GGAT, glutamate:glyoxylate aminotransferase; GDC, glycine decarboxylase complex; GLYK, glycerate kinase; GOX, glycolate oxidase; HPR, hydroxypyruvate reductase; PGLP, 2-PG phosphatase; PLGG1, plastidial glycolate/glycerate transporter 1; Rubisco, RuBP carboxylase/oxygenase; RuBP, ribulose-1,5-bisphosphate; SGAT, serine:glyoxylate aminotransferase; SHMT, serine hydroxymethyltransferase; THF, tetrahydrofolate; MAMP, microbe-associated molecular pattern; DAMP, damage-associated molecular pattern; PRR, pattern recognition receptor; NLR, nucleotide-binding/leucine-rich-repeat receptor; PTI, pattern-triggered immunity; ETI, effector-triggered immunity; ROS, reactive oxygen species; SA, salicylic acid; JA, jasmonate.

In nature, plants are constantly exposed to a dynamic external biotic environment, which drives the development of the plant immune system. As the first layer of immunity, elicitors from pathogenic and nonpathogenic microbes, known as microbe-associated molecular patterns (MAMPs), are recognized by plasma membrane-localized receptors known as pattern recognition receptors (PRRs) to activate pattern-triggered immunity (PTI) (Yu et al., 2017). Flg22, a peptide from the conserved domain of the bacterial flagellin, is one of the MAMPs. PTI also comprises plant responses to plant-derived endogenous elicitors generated in response to wounding or infection, such as small peptides and nucleotides, which are called damage-associated molecular patterns (DAMPs) (Yu et al., 2017). During PTI, intracellular signaling, transcriptional reprogramming, and other physiological responses culminate to limit pathogen growth. These events include increases in cytosolic Ca^2+^ concentration, reactive oxygen species (ROS) burst, and biosynthesis of phytohormones such as salicylic acid (SA) and jasmonate (JA) (Yu et al., 2017). To infect successfully, most pathogens can secrete virulent effectors into plant cells to suppress plant defense (Deslandes and Rivas, 2012). As the second layer of immunity, plants use intracellular nucleotide-binding/leucine-rich-repeat (NLR) receptors to recognize effectors, either directly or indirectly, leading to the activation of effector-triggered immunity (ETI) (Cui et al., 2015). ETI responses are similar to, but stronger than, those of the PTI, and often cause local programmed cell death called the hypersensitive response (HR) (Cui et al., 2015) (**Figure 1**). Recent studies reveal that PTI and ETI are not simply two independent and distinct pathways but work together to regulate immune responses (Ngou et al., 2021; Pruitt et al., 2021; Yuan et al., 2021).

Photorespiration is one of the numerous cellular pathways shown to be involved in immune response. Closely linked to photosynthesis, photorespiration is initiated by the oxygenation of ribulose 1,5-bisphosphate (RuBP) catalyzed by ribulose 1,5-bisphosphate carboxylase-oxygenase (Rubisco), producing 2-phosphoglycolate (2-PG) which can inhibit cellular functions when accumulated. 2-PG is first dephosphorylated by 2-PG phosphatase (PGLP) to produce glycolate, which is then transported out of the chloroplast by plastidial glycolate/glycerate transporter 1 (PLGG1) and bile acid sodium symporter 6 (BASS6). Upon entering the peroxisome, glycolate is converted to glyoxylate by glycolate oxidase (GOX), producing H_2_O_2_ that is then scavenged by catalase (CAT). Both glutamate:glyoxylate aminotransferase (GGAT) and serine:glyoxylate aminotransferase (SGAT) catalyze the conversion of glyoxylate to glycine. After transporting to the mitochondrion, glycine is converted to serine by the glycine decarboxylase complex (GDC) and serine hydroxymethyltransferase (SHMT), accompanying the tetrahydrofolate (THF) cycle and releasing CO_2_ and NH_3._ Serine is then transported back to the peroxisome, converted to hydroxypyruvate by SGAT, and subsequently to glycerate by hydroxypyruvate reductase 1 (HPR1). HPR2 is an HPR isoform that can reduce hydroxypyruvate to glycerate in the cytosol. Finally, glycerate is imported into the chloroplast through PLGG1 and phosphorylated to 3-phosphoglycerate (3-PGA) by glycerate kinase (GLYK) to recycle back to the Calvin-Benson cycle. Photorespiration consumes ATP in the chloroplast and NAD(P)H in the peroxisome and the cytosol, and releases NADH in the mitochondrion (Eisenhut et al., 2019) (**Figure 1**).

Although photorespiration significantly reduces photosynthetic efficiency (Walker et al., 2016), it is essential to C3 plants and even vital for C4 plants such as maize (Zelitch et al., 2009) and *Flaveria bidentis* (Levey et al., 2019), highlighting its importance to plant survival. The plant immune system appears to take advantage of photorespiration as well. For example, tightly connected with plant primary metabolism (Shi and Bloom, 2021), photorespiration can provide signals, substrates, or energy for immunity in face of pathogen invasion. In addition, the coupled response of photorespiration to environmental signals like dynamic light intensities and stomatal conductance (Fu and Walker, 2023) may represent a way for immunity to integrate environmental cues for optimal response.

At present, no unequivocal conclusions have been drawn on how the level of photorespiratory enzymes is regulated in response to pathogen infections. Some studies show that photorespiratory genes are generally suppressed by pathogen infection (Zabala et al., 2015; Giraldo – González et al., 2021; Kalapos et al., 2021; Yue et al., 2021), whereas in other studies certain photorespiratory genes show increased expression instead (Mitsuya et al., 2009; Ahammed et al., 2018). At the protein level, both up- and down-regulation of the photorespiratory enzymes in presence of pathogens have been observed (Segarra et al., 2007; Zhao et al., 2013; Ma et al., 2020; He et al., 2021). These discrepancies are likely due to the different plant-pathogen systems used and may indicate the complex nature of the response of various photorespiratory genes to stress at the expression and protein levels.

Although the importance of photorespiratory ROS in defense is supported by abundant evidence (Sørhagen et al., 2013), how photorespiration fully participates in immunity remains an intriguing question. Evidence also exists to support the notion that photorespiration is involved in immunity *via* other mechanisms, such as through photorespiratory metabolites and defense hormone biosynthesis. Here, we summarize available evidence showing the connection between photorespiration and immunity and discuss current understanding of the underlying mechanisms.

## Photorespiratory ROS: Important players in immune response

ROS such as H_2_O_2_ are crucial signaling molecules during plant-pathogen interactions (Camejo et al., 2016). Photorespiration is a major source of H_2_O_2_ in photosynthetic cells (Foyer et al., 2009), and photorespiratory organelles such as peroxisomes also contain H_2_O_2_-scavenging systems such as catalases (see below). Not surprisingly, studies of the roles of photorespiration in plant immunity have been mainly focused on H_2_O_2_.

GOX (**Figure 1**) contributes to disease resistance through its H_2_O_2_-producing capability. *GOX*-silenced tobacco plants show compromised non-host resistance to bacterial pathogens *Pseudomonas syringae* pv*. tomato* (*Pst*) strain T1, *P. syringae* pv*. glycinea* and *Xanthomonas campestris* pv. *vesicatoria*, as well as reduced ETI responses to the effector AvrPto (Rojas et al., 2012). Consistently, *GOX*-deficient Arabidopsis mutants show compromised non-host resistance to *P. syringae* pv*. syringae* strain B728A and *P. syringae* pv*. tabaci*, and reduced ETI responses to the effectors AvrB and AvrRps4 (Rojas et al., 2012). Null mutants of HAOX (hydroxy-acid oxidase), the enzyme that belongs to the same L-2-HAOX family as GOX (Esser et al., 2014), exhibit *gox*-like phenotypes in response to pathogens (Rojas et al., 2012). The Arabidopsis *gox* and *hoax* mutants also have decreased H_2_O_2_ levels after *P. syringae* pv. *tabaci* infection, which is independent of the H_2_O_2_-producing enzyme, NADPH oxidase (Rojas and Mysore, 2012; Rojas et al., 2012). In addition, reducing *GOX2* expression in tomato lowers H_2_O_2_ levels in the leaf and increases plant susceptibility to the compatible pathogen *Pst* DC3000, a phenotype that can be rescued by H_2_O_2_ pre-treatment (Ahammed et al., 2018). Similarly, decreases in the level of H_2_O_2_ and increases in *Pst* DC3000 susceptibility were seen after application of isonicotinic acid hydrazide (INH), an inhibitor that blocks the conversion of glycine to serine in photorespiration and suppresses GOX activity (Ahammed et al., 2018). These results suggest that the H_2_O_2_ produced by GOX family members is important to immunity. However, silencing *GOX1* in rice results in enhanced resistance to the compatible pathogen *X. oryzae* pv. *oryzae* (Chern et al., 2013). Additionally, three members from the tobacco GOX family contribute differently to H_2_O_2_ levels and defense (Xu et al., 2018), yet all five members of the Arabidopsis GOX family work additively to increase resistance (Rojas et al., 2012). These inconsistent results regarding the function of different GOX members may be due to distinct plant-pathogen systems utilized and the functional divergence of family members in different plant lineages.

The function of the H_2_O_2_-scavenging enzyme CAT (**Figure 1**) in immune response has been investigated extensively. Without pathogen infection, CAT-deficient mutants show SA accumulation, induced expression of the SA-pathway marker gene *PR1* (pathogenesis-related 1), cell death, along with H_2_O_2_ accumulation in tobacco (Takahashi et al., 1997; Chamnongpol et al., 1998; Mittler et al., 1999) and Arabidopsis (Chaouch and Noctor, 2010; Chaouch et al., 2010). In addition, SA was found to bind to CAT and inhibit CAT activity to increase the level of H_2_O_2_ in a variety of plant species (Chen et al., 1993; Sánchez-Casas and Klessig, 1994). The inhibition of CAT activity by SA analogs correlates with the induction of the *PR1* gene and plant resistance to tobacco mosaic virus (Conrath et al., 1995). Suppression of CAT2 by SA in Arabidopsis also leads to decreases in auxin and JA biosynthesis (Yuan et al., 2017). This is consistent with the increased biotroph resistance that is dependent on SA and repressed by auxin, and decreased JA-dependent necrotroph resistance in the *cat2* mutant (Yuan et al., 2017). This data supports the role of CAT2 as a mediator between SA and auxin/JA signaling pathways in response to different pathogens. CAT2 also seems to connect Ca^2+^ signaling to the JA pathway, as the calmodulin-binding protein IQM1 (IQ-Motif Containing Protein 1) positively regulates JA biosynthesis by enhancing CAT2 function at both the transcription and enzymatic activity levels (Lv et al., 2019). The transcription factor GBF1 (G-box binding factor 1) downregulates *CAT2* expression during pathogen response, leading to high H_2_O_2_ levels (Giri et al., 2017), reinforcing the view that photorespiratory H_2_O_2_, whose level is modulated by CATs, may act as a hub in coordinating defense responses.

Moreover, pathogens often target CAT to help with infection, which also suggests the importance of photorespiratory H_2_O_2_ in immunity. Effectors from the bacterial pathogen *Ralstonia solanacearum* (Sun et al., 2017) and the root-knot nematode *Meloidogyne incognita* (Zhao et al., 2021) inhibit CAT activity *via* physical interaction with the enzyme, and the 2b protein from the *Cucumber mosaic virus* induces CAT3 degradation in Arabidopsis (Murota et al., 2017). However, some pathogens seem to regulate the level of CAT positively. For example, the *Pepino mosaic virus* utilizes Triple Gene Block Protein 1 (TGBp1) to promote the activity of CAT1 and reduce H_2_O_2_ levels in tomato (Mathioudakis et al., 2013). Interestingly, the oomycete pathogen *Phytophthora sojae* has two effectors that interact with CATs and regulate H_2_O_2_ homeostasis in opposite directions (Zhang et al., 2015).

Evidence suggesting that CAT and GOX act together to regulate H_2_O_2_ homeostasis in defense has been reported. Under sub-ambient CO_2_ conditions, enhanced resistance to the biotrophic oomycete *Hyaloperonospora arabidopsidis* and high intracellular ROS content were observed in Arabidopsis (Williams et al., 2018). This resistant phenotype is abolished in the *gox1* or *haox1* mutants under the same low CO_2_ conditions after pathogen inoculation, and the *CAT2* gene is down-regulated by infection (Williams et al., 2018), suggesting that both boosted GOX and suppressed CAT contribute to ROS accumulation. More direct evidence comes from rice, where SA treatment disrupts the physical interaction between GOX and CAT and induces H_2_O_2_ accumulation (Zhang et al., 2016). These results suggest that H_2_O_2_ homeostasis during plant-pathogen interaction is possibly regulated by the association and disassociation of GOX and CAT.

Besides peroxisomal H_2_O_2_, mitochondrial ROS can be influenced by photorespiration and involved in defense as well. The P-protein and H-protein of GDC, the mitochondrial multienzyme complex that catalyzes glycine decarboxylation (**Figure 1**), are repressed in activity by the victorin toxin produced by the fungus *Cochliobolus victoriae* (Navarre and Wolpert, 1995). Victorin treatment triggers mitochondrial ROS burst and subsequent apoptotic response in oat, a similar result to that caused by the GDC inhibitor aminoacetonitrile (AAN) (Yao et al., 2002). In addition, silencing *GDC-T* or *GDC-P* in tobacco suppresses victorin-triggered cell death and ETI response to the effector AvrPto (Gilbert and Wolpert, 2013). Furthermore, the bacterial elicitor harpin also inhibits GDC activity in Arabidopsis, resembling the inhibition by AAN treatment (Cristina Palmieri et al., 2010). Therefore, it is likely that GDC plays a role in reducing the level of ROS during plant-pathogen interaction to avoid damages caused by excess ROS.

The peroxisomal aminotransferase GGAT, which converts glyoxylate to glycine (**Figure 1**), is also connected with H_2_O_2_. Compared to wild-type plants, the Arabidopsis *ggat1* mutant is more resistant to the necrotrophic fungal pathogen *Botrytis cinerea* and contains lower H_2_O_2_ concentrations upon infection, whereas a higher H_2_O_2_ level is observed when uninfected (González-lópez et al., 2021). How GGAT regulates H_2_O_2_ and whether this change in H_2_O_2_ levels imposes significant impacts on immune responses remains unknown.

The impact of photorespiration on ROS levels may differ among the three photorespiratory organelles during plant-pathogen interactions. In the chloroplast, photorespiration may actually prevent ROS production during plant immune response. As the major source of chloroplastic ROS, the photosynthetic electron transport chain produces excessive reducing equivalents and ATP under stress conditions (Voss et al., 2013). Therefore, photorespiration may function as an alternative sink for these reducing equivalents and ATP to decrease ROS accumulation in the chloroplast and protect photosystems from photodamage (Voss et al., 2013). Meanwhile, it is likely that the high photorespiratory rate under stress conditions enhances H_2_O_2_ production in the peroxisome, and increases NADH production by GDC in mitochondria to increase the level of mitochondrial ROS. Nonetheless, these hypotheses remain to be tested under pathogen defense conditions.

In conclusion, extensive evidence has demonstrated the key roles of ROS in plant immune response. The level of H_2_O_2_ is impacted by photorespiratory enzymes such as GOXs and CATs in peroxisomes and GDC in mitochondria, and potentially other photorespiratory proteins as well.

## Involvement of photorespiratory metabolites in immunity

Photorespiration involves a variety of metabolites connected to several primary metabolic pathways, including photosynthesis, C_1_ metabolism, amino acid metabolism, and nitrogen assimilation (Hodges et al., 2016). Metabolite analysis of Arabidopsis suspension cultured cells in which immunity was activated by *Pst* DC3000, mutant *Pst* DC3000 (D28E), or flg22, revealed large-scale metabolic changes, including the glyoxylate and dicarboxylate metabolism and the amino acid metabolism that partially overlap with the photorespiratory pathway (Misra et al., 2016). In cucumber, nitrate-induced resistance to the fungus *Fusarium oxysporum* f. sp. *cucumerinum* (FOC), along with the accumulation of most of the photorespiratory intermediates except serine, was observed (Sun et al., 2021). As discussed below, specific photorespiratory metabolites have also been shown to be involved in plant-pathogen interactions.

Catalyzing the bidirectional conversion of serine and THF to glycine and 5,10-methylene-THF, the photorespiratory enzyme SHMT (**Figure 1**) is also a crucial enzyme in C_1_ metabolism (Hanson and Roje, 2001). *GmSHMT08c*, which encodes a cytosolic SHMT in soybean, was identified to be a resistant gene to the soybean cyst nematode (*Heterodera glycines*, SCN) (Liu et al., 2012; Kandoth et al., 2017). The resistance is resulted from two amino acid substitutions in the GmSHMT08c protein that impede THF binding and reduce catalytic activity of the enzyme (Liu et al., 2012; Korasick et al., 2020). GmSHMT08c confers SCN-resistance in soybean roots (Liu et al., 2012), so it is less likely that photorespiration is involved in this resistance. Other members of the GmSHMT family do not seem to function in SCN resistance individually (Lakhssassi et al., 2019). However, considering the probable functional redundancy of the five mitochondrial GmSHMT members, folate metabolism is a possible point at which photorespiration affects plant immunity. Moreover, the Arabidopsis *shmt1* mutant exhibits compromised defense responses to both biotrophic and necrotrophic pathogens (Moreno et al., 2005). Silencing tomato *SHMT1* dampens resistance to *P. syringae* independent of H_2_O_2_, whereas overexpressing the gene enhances the resistance (Ahammed et al., 2018). Further, Arabidopsis SHMT4 binds to SA (Manohar et al., 2015), and rice SHMT1 interacts with the disease-resistance protein RPM1 (Wang et al., 2021), although their roles in immunity in these contexts have not been shown. Taken together, SHMT plays a role in defense response in several plant species. Except for the potential connection to folate metabolism in soybean, the underlying mechanisms are still unknown in most species.

The peroxisomal HPR enzyme that converts hydroxypyruvate to glycerate (**Figure 1**) engages in immunity through photorespiratory metabolites. A soybean HPR interacts with P34, the receptor of the *P. syringae* elicitor syringolide, and applying glycerate and 3-PGA, products of the HPR-catalyzed reaction and the downstream step, respectively, restrains syringolide-triggered HR (Okinaka et al., 2002). Additionally, the cytosolic Arabidopsis HPR2 protein binds to SA, but evidence for its role in immunity is lacking (Manohar et al., 2015).

The role of photorespiration-associated amino acids in plant immunity has been illustrated in several studies. In rice, 18 different amino acids, among which glutamate, glycine and serine are photorespiratory intermediates (**Figure 1**), can induce systemic resistance against rice blast when individually applied to roots (Kadotani et al., 2016). Soaking tomato fruits in glutamate solution reduces colonization of the fungal pathogen *Alternaria alternata* and activates several primary metabolic pathways such as nitrogen metabolism, the γ-aminobutyric acid shunt, and SA signaling (Yang et al., 2017). Consistently, glutamate can serve as a DAMP to induce Ca^2+^ signaling and thereafter defense responses in plants (Toyota et al., 2018).

Taken together, current data provide evidence for the influence of photorespiratory metabolites on plant defense response. Further and in-depth studies are needed to elucidate the underlying mechanisms.

### Influence of photorespiration on the biosynthesis of defense hormones

SA and JA are the two major phytohormones in plant defense (Pieterse et al., 2012). SA is synthesized in plastids and in the cytosol (Lefevere et al., 2020), and the biosynthesis and activation of JA involve plastids, peroxisomes and the cytosol (Wasternack and Song, 2017). Recently, CAT2-promoted JA biosynthesis in Arabidopsis was shown to be achieved by the direct interaction between the N-terminus of CAT2 and the JA biosynthetic enzymes acyl-CoA oxidase 2 (ACX2) and ACX3, without the requirement of H_2_O_2_ (Zhang et al., 2021). Another study demonstrated that the JA-activated defense to the necrotrophic pathogen *Erwinia amylovora* is partially dependent on GOX2 and does not involve obvious changes to the level of H_2_O_2_ (Launay et al., 2022), indicating that other mechanisms independent of H_2_O_2_ may exist in this immune response. Given the overlap of the locations for photorespiration and defense hormone biosynthesis in several subcellular compartments, it is possible that one or multiple photorespiratory enzymes or metabolites serve as mediators or signals in the biosynthesis of SA and JA. Although evidence for the connection between photorespiration and defense hormone biosynthesis is still scarce, it is a promising research direction that merits further investigations.

## Other photorespiratory components involved in defense

A few other photorespiratory enzymes are also involved in immunity, yet the mechanisms behind are inconclusive.

In a *Pseudoperonospora cubensis*-resistant melon cultivar, genes encoding two aminotransferases — homologs of the Arabidopsis peroxisomal aminotransferase SGAT, which converts glyoxylate to glycine and serine to hydroxypyruvate (**Figure 1**), were found among the resistance genes (Taler et al., 2004). Overexpressing either gene confers resistance to the pathogen in the susceptible cultivar (Benjamin et al., 2009). That the resistant melon cultivar also exhibits high GOX activities indicates that this SGAT-regulated resistance may be attributed to high H_2_O_2_ levels (Taler et al., 2004). However, the positive role of SGAT in plant resistance to *P. syringae* in tomato was shown to be independent of H_2_O_2_ (Ahammed et al., 2018). Additionally, Arabidopsis SGAT was identified as an SA-binding protein, with unknown consequences in defense (Manohar et al., 2015). Further studies are needed to dissect the precise mechanism of the role of SGAT in immunity.

The chloroplast photorespiratory kinase GLYK, which phosphorylates glycerate to make 3-PGA (**Figure 1**), appears to play a positive role in immunity at multiple levels. Full-length GLYK in potato is a target for the Irish potato famine pathogen *Phytophthora infestans* effector protein AVRvnt1 through protein binding, resulting in the impediment of GLYK trafficking into chloroplasts and enhancement of GLYK degradation, as well as the activation of the ETI response mediated by Rpi-vnt1.1, the NLR that recognizes AVRvnt1 (Gao et al., 2020). *GLYK* silencing results in increased plant susceptibility to *P. infestans* lacking AVRvnt1 *via* an unknown mechanism (Gao et al., 2020). Interestingly, the full-length GLYK protein is mainly produced under the light (Gao et al., 2020), when photorespiration operates, indicating that the function of GLYK in immunity likely depends on photorespiration.

## Measurement of photorespiration rate in defense response

Measuring physiological parameters of photorespiration in plants after pathogen infection provides new perspectives in dissecting the relationship between photorespiration and defense. Photorespiration rate, which can be estimated by the difference of net CO_2_ assimilation rate between 2% and 21% O_2_, is increased upon *Pst* DC3000 infection, whereas INH, the inhibitor that blocks the conversion of glycine to serine in photorespiration and suppresses GOX activity, suppresses this increase (Ahammed et al., 2018). Other indicators of photorespiration rate used in the measurements include the photorespiratory CO_2_ compensation point (Γ*) and the ratio of glycine to serine (Gly/Ser). FOC-inoculated banana seedlings contain higher Γ* than untreated plants (Dong et al., 2016). In nitrate-induced FOC resistance cucumber plants, both Γ* and Gly/Ser are increased (Sun et al., 2021). Further studies are needed to determine whether the increased photorespiration rate reported in these studies contributes to defense responses. This quantitative approach may also be extended to additional studies aimed at dissecting the interplay between photorespiration and immunity.

## Discussion

The photorespiratory pathway has become a major target for genetic engineering with the goal to increase photosynthetic efficiency (Betti et al., 2016). Therefore, a more precise understanding of the contribution of photorespiration to plant physiology and plant interaction with the environment is vital for such efforts. Studies demonstrate the key role of photorespiration in plant immunity through changes in ROS homeostasis, while other mechanisms such as the participation of photorespiratory metabolites, the direct impact of photorespiration on defense hormone biosynthesis, and etc, are also emerging (**Table 1**). Considering the complexity of both the photorespiratory pathway and immune responses, large-scale and systematic approaches involving simultaneous measurements of photorespiration and immune response will be required in order to obtain a comprehensive view of the interplay between these two systems under different conditions. Such knowledge is highly needed for the rational design of a new generation of crop plants to feed the growing human population. Engineering photorespiration to manipulate ROS levels or the pool size of some metabolites may be a promising approach to enhance crop resistance.

**Table 1:** Photorespiratory enzymes that participate in defense response.

Enzyme	Full name	Function in immunity	References
**GOX**	Glycolate oxidase	Impacts ROS homeostasis and JA biosynthesis	Rojas and Mysore, 2012; Rojas et al., 2012; Chern et al., 2013; Zhang et al., 2016; Ahammed et al., 2018; Williams et al., 2018; Xu et al., 2018; Launay et al., 2022
**CAT**	Catalase	Impacts ROS homeostasis; suppressed by SA; promotes JA biosynthesis and mediates crosstalk between SA and JA/auxin, and between Ca^2+^ and JA (AtCAT2)	Chen et al., 1993; Sánchez-Casas and Klessig, 1994; Conrath et al., 1995; Takahashi et al., 1997; Chamnongpol et al., 1998; Mittler et al., 1999; Chaouch and Noctor, 2010; Chaouch et al., 2010; Mathioudakis et al., 2013; Zhang et al., 2015; Zhang et al., 2016; Giri et al., 2017; Murota et al., 2017; Sun et al., 2017; Yuan et al., 2017; Williams et al., 2018; Lv et al., 2019; Zhang et al., 2021; Zhao et al., 2021
**GGAT**	Glutamate: glyoxylate aminotransferase	Connects with H_2_O_2_	González-lópez et al., 2021
**SGAT**	Serine:glyoxylate aminotransferase	Positive role in resistance in melon and tomato; bound by SA (AtSGAT)	Taler et al., 2004; Benjamin et al., 2009; Manohar et al., 2015; Ahammed et al., 2018
**GDC**	Glycine decarboxylase complex	Impacts ROS homeostasis	Navarre and Wolpert, 1995; Yao et al., 2002; Cristina Palmieri et al., 2010; Gilbert and Wolpert, 2013
**SHMT**	Serine hydroxymethyl- transferase	Contributes to resistance possibly through folate metabolism; bound by SA (AtSHMT4); interacts with the disease-resistance protein RPM1 (OsSHMT1)	Moreno et al., 2005; Liu et al., 2012; Manohar et al., 2015; Kandoth et al., 2017; Ahammed et al., 2018; Lakhssassi et al., 2019; Korasick et al., 2020; Wang et al., 2021
**HPR**	Hydroxypyruvate reductase	Interacts with syringolide receptor (GmHPR); bound by SA (AtHPR2)	Okinaka et al., 2002; Manohar et al., 2015
**GLYK**	Glycerate kinase	Positive role in resistance in potato	Gao et al., 2020

## Author contributions

XJ and JH co-conceptualized this review. XJ, BW, SH, and JH co-wrote the manuscript. All authors contributed to the article and approved the submitted version.
